# Lameness and its relationship with health and production measures in broiler chickens

**DOI:** 10.1017/S1751731119000466

**Published:** 2019-03-21

**Authors:** E. G. Granquist, G. Vasdal, I. C. de Jong, R. O. Moe

**Affiliations:** 1 Faculty of Veterinary Medicine, Norwegian University of Life Sciences, Ullevålsveien 72, 0454 Oslo, Norway; 2 Norwegian Meat and Poultry Research Centre, Lørenveien 38, 0515 Oslo, Norway; 3 Wageningen Livestock Research, Wageningen University & Research, PO Box 338, 6700 AH, Wageningen, The Netherlands

**Keywords:** poultry, dermatitis, condemnation, gait, welfare

## Abstract

The aim of this study was to explore lameness and the associations between lameness and health/production measures of animal welfare in commercial broiler production, using the Welfare Quality^®^ protocol for broilers. A total of 50 flocks were included in the sample and farm visits were conducted for lameness scoring at a mean age of 28.9 days. The percentage of animals (*n*=7500) in the six different gait score (GS) categories were GS0: 2.53%, GS1: 44.19%, GS2: 33.84%, GS3: 16.32%, GS4: 2.36% and GS5: 0.53%. Production and other welfare data were collected for each flock after slaughter. Higher gait scores were associated with increased hock burn score (*P*<0.02), increased footpad dermatitis score (*P*<0.01), reduced bird cleanliness score (*P*<0.01) and peat litter (*P*<0.01). Although not statistically significant, there was a tendency for increased flock gait score being associated with wet litter (*P*=0.07). In addition, condemnations at *postmortem* inspection were associated with increasing gait scores (*P*<0.05), indicating that at least a portion of the lameness cases display pathological changes on the carcasses. In conclusion, 19%of the birds showed moderate-to-severe lameness, which was associated with several production or health and welfare observations including feather cleanliness and condemnations as unfit for human consumption at slaughter. Although stocking density and growth rate are already known key factors for lameness, associations of lameness with hock burns, footpad dermatitis and cleanliness of the birds suggest that a suboptimal physical environment (e.g. litter- and air quality) may be detrimental to leg health. Further studies are needed to explore these associations in more detail.

## Implications

Lameness in broiler chickens is a primary welfare concern as it is considered painful. Reduced growth and culling of lame birds also affects farm profitability. Footpad dermatitis may cause lameness and has been used as an indicator of welfare in chickens. Identifying risk factors associated with lameness (such as pathological conditions associated with condemnation at *postmortem* inspection) may provide important tools for flock welfare assessment.

## Introduction

There has been a perpetual concern for the welfare of chickens in modern broiler production, especially regarding lameness (Julian, 1998; EFSA, 2012; Kapell *et al.*, 2012). Although high growth rate is identified as a key factor for lameness (Julian, 1998; Kapell *et al.*, 2012), factors such as stocking density, diseases, nutritional deficiencies, air quality, light, circadian rhythms, age, BW, genetics and management practices are also known to be associated (Julian, 1998; Sørensen *et al.*, 2000; Williams *et al.*, 2000; Bradshaw *et al.*, 2002; Bessei, 2006; Knowles *et al.*, 2008). Lameness in broilers is usually assessed by examining the gait of individual birds using, for example, the Bristol gait scoring system, which scores from 0 (normal) to 5 (unable to walk) (Kestin *et al.*, 1992). Several studies found that 14% to 50% of broilers suffer from lameness as reflected by gait scores 3, 4 or 5 (Kestin *et al.*, 1992; Sanotra *et al.*, 2003; Knowles *et al.*, 2008; de Jong *et al.*, 2011; Bassler *et al.*, 2013; Kittelsen *et al.*, 2017). Lameness is associated with pain (McGeown *et al.*, 1999), therefore representing an important welfare concern. Indeed, studies have shown that lame birds (gait score ⩾3) prefer food with analgesic, and that lame broilers increase their activity when given analgesics (McGeown *et al.*, 1999; Danbury *et al.*, 2000; Caplen *et al.*, 2013; Hothersall *et al.*, 2016). Furthermore, lame birds may have more difficulties reaching resources in the house such as food and water (Weeks *et al.*, 2000; Butterworth *et al.*, 2002; Sanotra *et al.*, 2002). Lameness is also related to difficulties in escaping aversive encounters, and in performing behaviour such as dust bathing (Vestergaard and Sanotra, 1999), foraging, walking and preening (Weeks *et al.*, 2000), thereby further compromising their welfare. Lameness has been shown to negatively affect the final slaughter weight (Gocsik *et al.*, 2014) and increased lameness has also been associated with a higher mortality in flocks (Wideman *et al.*, 2012), thus also having a negative impact on the farmer’s economy. Previous studies suggest that infectious leg disorders such as bacterial chondronecrosis and osteomyelitis may be important underlying causes for lameness (Bradshaw *et al.*, 2002; Kittelsen *et al.*, 2015), and a recent study found that impaired gait in broilers close to slaughter age was associated with increased 1^st^ week mortality (Kittelsen *et al.*, 2017). This association between 1^st^ week mortality and later lameness indicates that early infections in the day-old chick may be implicated and such infections may eventually cause lameness. First week mortality can, however, also have non-infectious causes such as dehydration and starvation. It is unclear whether potential persistent infections are associated with other flock-based welfare issues, or related to health and production.

The aim of this study was to investigate associations between lameness and commonly used health- and production-related measures of animal welfare in broiler flocks. We hypothesized that there is a relationship between lameness and the general health status of the broiler chicken flocks, which may influence production measures.

## Material and methods

### Study design

In total, 50 commercial broiler chicken farms were selected from the list of about 150 broiler producers delivering chickens (hybrid: Ross 308, mixed sex) to a slaughter plant, located in the southeast of Norway (Nortura Hærland). The producers were contacted by phone a few weeks before the visit. Participation in the study was voluntary. All enrolled farms were visited during January to March 2015. Each flock was assessed according to the Welfare Quality^®^ (2009) protocol for broilers. The flock sizes in the observed flocks ranged from 3900 to 28 900 birds, and the stocking density ranged from 15 to 33 kg/m^2^. Table 1 displays some flock characteristics of the sampled flocks. Each flock was examined on the farm by the same observer between 28 and 30 days of age (average age of slaughter in Norway is 31 days). The individual farms received their day-old chickens from one of three different hatcheries out of which one served 44 of the 50 flocks. Data from the meat inspection and production data were collected from the abattoir for each flock, shortly after processing. Meat inspection was performed in accordance with EU regulation (Comission Regulation (EC) No. 854/2004, 2004). The sub-categories of condemnations at meat inspection were as follows: omphalitis, circulatory disorders, liver lesions, ascites, abnormal growth, wounds and abnormal colour and odour. In addition, data on condemnations due to technical injuries or faecal contamination were collected, but not used in the further data analyses. The categories of condemnation are only crude indications of pathological conditions and do not involve necropsy examination performed by pathologist.

**Table 1:** Descriptive broiler flock data

Measure (continuous)	*N*	Mean	SD	Min	Max
Broilers in flock (*n*)	50	17391.06	6080.25	3900	28950
Age parent flock (weeks)	48	37.35	6.29	27	51
Age at visit (day)	50	28.90	1.80	27	34
Live weight at visit (g)	50	1587.54	231.75	1075	2500
Live weight at slaughter (g)	49	1861.43	197.43	1631	2513
Daily weight gain (g)^a^	50	40.18^a^	2.76	35.10	48.23
FCR (kg weight/kg feed)	50	2.16	0.08	2.02	2.41
Footpad dermatitis score (0 to 200)	50	15.54	22.38	0	111
Hock burn score (0 to 200)	50	5.78	11.77	0	53
Cleanliness score (0 to 300)	50	97.54	15.50	6	115
Flock litter quality scores (0 to 5)^b^	50	2.23	1.04	1	4.8
Birds per drinker (*n*)	50	16.74	5.45	6.69	33
Total mortality (%)	50	2.20	0.83	1.14	5.39
Culled (% of total mortality)	35	25.15	11.89	8.33	55.55
Stocking density (animals/m2)	50	17.42	2.55	9.14	20.55
Stocking density (kg/m2)	50	27.33	3.83	15.54	33.19
Flock weight uniformity (CV)	50	0.14	0.04	0.11	0.26
Measure (ordinal)	*N*	Median	Interquartile range	Min	Max
Individual gait score (0 to 5)	7500	2	1 to 2	0	5
Litter quality measurements (0 to 5)	281	2	1 to 3	0	5
Level of dust (0 to 4)	50	2	2 to 3	1	4

FCR=feed conversion rate; CV=CV (%/100).

a Daily weight gain calculated at slaughter.

b Mean of several measurements in each house.

### Farm visits

For detailed description of welfare assessments, we refer to the Welfare Quality^®^ (2009) broiler assessment protocol. Before the start of the study, the observer received training by experienced persons in the theory and practise of the protocol. Each farm visit was completed within 3 to 4 h. During every farm visit, the observer used a new dark-blue overall with a hood and plastic boots. Data from the farm visits were recorded on the site, using specialized software on a personal digital assistant (PDA). (Software designed by H. van den Heuvel, Wageningen University and Research, Wageningen Livestock Research.)

The assessment started with an initial consultation with the farmer, where information such as number of animals originally placed, hatchery, age of parent flock, house dimensions, litter type, feed type, mortality and number of culled animals was recorded. Then, the assessments continued according to the Welfare Quality^®^ protocol for broilers. Results on the touch test are reported elsewhere (Vasdal *et al.*, 2018) and will not be discussed here.

To assess lameness in the flock, 150 birds from at least five different and arbitrary locations in the house were gait scored as follows: at each location, around 30 birds were carefully fenced in, using a mobile cardboard catching pen that was placed around a group of animals with minimal disturbance. The five locations were selected to avoid repeated assessments. Each bird was individually encouraged to walk out of the pen and then scored. Gait scores were classified according to these criteria: (0) Normal, dexterous and agile. (1) Slight abnormality, but difficult to define. (2) Definite and identifiable abnormality. (3) Obvious abnormality, affects ability to move. (4) Severe abnormality, only takes a few steps. (5) Incapable of walking (Kestin *et al.*, 1992).

After the gait scoring was completed, a total of other 100 birds in five different locations were scored for plumage cleanliness (scored from 0 (clean) to 3 (feathers very dirty)), footpad dermatitis (scored from 0 (no footpad lesion) to 4 (severe lesion, large area injured)) and hock burns (scored from 0 (no hock burn) to 4 (severe, dark coloured lesion of considerable size)). In addition, several resource-based measures such as litter quality (scored from 0 (completely dry) to 4 (stick to boot once the crust is broken)) at minimum four and maximum six different locations in the house, level of dust (scored from 0 (no dust) to 4 (thick layer of dust)), drinker types and drinker space (birds per drinker) were recorded. According to the Welfare Quality^®^ (2009) broiler assessment protocol, mean dust- and litter scores for each broiler house were used in the analyses.

### Calculation of scores

Gait score for each flock was calculated by multiplying all animals with score 0 with 0, all animals with score 1 with 1 and so on for 150 scored animals in each flock: ∑=((*n*0×0)+(*n*1×1) +(*n*2×2)+(*n*3×3)+(*n*4×4)+ (*n*5×5)). The total flock gait score could theoretically range from 0 (all 150 animals receive score 0) to 750 (all 150 animals receive score 5). Thus, an increased flock gait score indicates increased lameness, but a few severely lame birds in a flock could give a similar score as a flock with many, but only moderately lame birds.

Footpad dermatitis and hock burn scores were calculated by multiplying all animals with score 0 with 0, all animals with scores 1 and 2 with 1, and animals with scores 3 and 4 with 2: Ʃ=(*n*0×0)+((*n*1+*n*2) ×1)+((*n*3+*n*4) ×2). The total flock score could theoretically range from 0 (all 100 animals receive score 0) to 200 (all 100 animals receive scores of 3 and 4), which is the same procedure commonly used at the slaughterhouses. Cleanliness score was calculated by multiplying all animals with score 0 with 0, all animals with score 1 with 1 and so on for all 100 animals: Ʃ=((*n*0×0)+(*n*1×1)+(*n*2×2)+(*n*3×3)). The total flock cleanliness score could theoretically range from 0 (all 100 animals receive score 0) to 300 (all 100 animals receive score 3). Thus, an increased cleanliness score indicates soiled birds.

### Statistical methods

The data were collected on a handheld computer at the farm and transferred to an Excel (2013) spreadsheet and further to Stata SE 14 (Stata Corp LP, TX, USA). Inspection of the variables were performed in Stata using graphical tools (box plots, histograms and scatter diagrams), tabulations, calculations of means, medians, interquartile ranges, standard errors and 95% confidence intervals. Gait score was the outcome of the analyses and was considered normally distributed after log10 transformation. Univariable linear regression was used to study the effects of independent variables on the flock level gait score. Independent variables that obtained a *P*-value of <0.2 in univariable association with the dependent variable, were included in a multivariable regression model. The final model was obtained by backward exclusion until all independent variables obtained a *P*-value of <0.05 in the model. The multivariable regression model used the transformed (log10) variable of gait scores and the output of the model displays the logarithmic association. Residuals of univariable and multivariable regressions were inspected for normality by normal quantile plots. The final model was derived, based on information criteria analyses and likelihood ratio tests for every inclusion or exclusion of predictors in the model. No collinearity or interactions were revealed by the analyses.

## Results

### Descriptive flock data

Table 1 shows descriptive data of the study population and comprise of flock characteristics, production data, health parameters and production environmental data.

Table 2 shows the frequency of causes for condemnations at the *postmortem* inspection. These results were used as an indication of the health status of the study population. The most common causes of condemnation as unfit for human consumption were circulatory disorders and ascites.

**Table 2:** The mean total condemnation in the broiler flocks and the proportions (P) of causes of condemnation as unfit for human consumption

Measure	*N*	Mean	Median	Min	Max	SD
Total condemnation (%)	50	0.89	0.82	0.36	2.61	0.45
Subcategories^**a**^	*N*	P	Median	Min	Max	SD
Abnormal colour or odour (%)	50	0.03	0.02	0.00	0.18	0.03
Omphalitis (%)	50	0.03	0.02	0.00	0.27	0.04
Circulatory disorders (%)	50	0.44	0.36	0.00	2.97	0.44
Liver lesions (%)	50	0.05	0.02	0.00	1.10	0.15
Ascites (%)	50	0.26	0.20	0.00	1.65	0.27
Abnormal growth (%)	50	0.09	0.05	0.00	1.76	0.25
Wounds (%)	50	0.06	0.03	0.00	1.10	0.17
Other (%)	50	0.04	–	–	–	–

a Data obtained from the Norwegian Food Authority.

### Lameness

**Figure 1:**
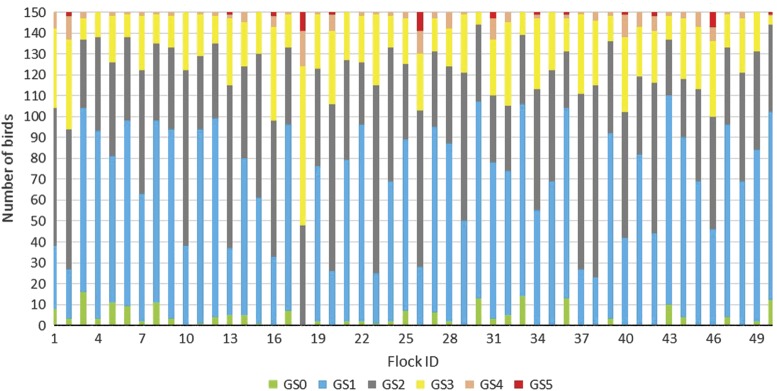
Number of broilers (n=7500) in each of the six gait scoring categories (0 to 5) in each of the 50 farms (n=150 birds per farm).

Table 3a shows the distribution of gait scores in the study population. The mean age at scoring was 28.9 days. Three per cent% of the study sample had a normal gait (score 1), whereas 19% had a score at 3 or above, representing an obvious abnormality, severe abnormality or incapability of walking. Birds with moderate-to-severe lameness (i.e. gait score ⩾3) were found in all flocks; however, the prevalence of such birds varied substantially between flocks. Table 1 shows the median gait score from individual gait scoring and Table 3b shows the mean gait scores in the study sample. The flock level gait score ranged from 186 to 439, with an average of 259.4±52.02 (Table 3b). Figure 1 shows the grading of individual birds and allocation to each gait score category (0 to 5).

**Table 3:** Distribution of broilers (*n*=7500) within the different gait scoring categories

A: Distribution of gait scores in the total sample	Proportion	SD	Min	Max
0 – normal, dexterous and agile	0.03	0.03	0	0.10
1 – slight abnormality, but difficult to define	0.44	0.18	0	0.67
2 – definite and identifiable abnormality	0.34	0.12	0.18	0.62
3 – obvious abnormality, affects ability to move	0.16	0.08	0.03	0.50
4 – severe abnormality, only takes a few steps	0.02	0.03	0	0.11
5 – incapable of walking	0.01	0.01	0	0.06
B: Mean individual and flock level scores in sample	Mean score	SD	Min	Max
0 to 750 – total gait score on flock level	259.40	52.03	186	439

Table 4 shows the univariable associations between flock-level gait scores and health- and production-related measures of welfare. The analyses revealed that there were associations between gait score and condemnations (unfit for human consumption) at the meat inspection. The association indicates that one log increase in gait score at the flock level, results in a 0.052 log increase in the percentage of condemned broiler carcasses (*P*<0.05). A subcategory of condemnations; abnormal colour and odour is associated with abnormal gait for which one log unit increase results in 0.74 log increase in condemnations (*P*<0.05). Further, abnormal gait was associated with hock burns (*P*<0.005), foot pad dermatitis (*P*<0.01) and cleanliness score of the bird (*P*<0.01). Although not statistically significant, there was a tendency for increased flock gait score being associated with wet litter (*P*=0.07). The multiple regression model retained significant relationships between five different predictors and the flock-level gait score (Table 5). The model found significant associations between gait score and type of litter (*P*<0.01), weight of birds at farm visit (*P*<0.05), total condemnations (*P*<0.01) at meat inspection, hock burns (*P*<0.01) and cleanliness score (*P*<0.01). The coefficient of multiple determination was 0.59, indicating a good fit of the model to the observed data.

**Table 4:** Univariable associations with the mean of individual gait scores on broiler flock level

Gait score (log10)	Coefficient log10(*y*)	SEM	*z* ^a^	*P*>|*z*|	95% CI^b^
Peat litter *v*. wood shavings	0.074	0.042	1.75	0.087	−0.011, 0.158
Litter quality (0 to 5)	0.021	0.011	1.87	0.068	−0.002, 0.043
Stocking density (kg/m^2^)	−0.001	0.003	−0.18	0.857	−0.007, 0.006
Live weight at visit (g)	−0.000	0.000	−1.75	0.086	−0.000, 0.000
Live weight at slaughter (g)	0.000	0.000	1.34	0.186	−0.000, 0.000
Daily weight gain (g/day)	0.004	0.004	0.88	0.382	−0.005, 0.012
Total mortality (%)	−0.003	0.014	−0.22	0.827	−0.032, 0.026
1^st^ week mortality (%)	−5.060	3.057	−1.66	0.105	−11.214, 1.094
Culled (% of total mortality)	0.000	0.001	0.27	0.786	−0.002, 0.003
Total condemnation (%)	0.052	0.025	2.05	0.045	0.001, 0.104
Abnormal colour and odour (%)	0.738	0.352	2.09	0.042	0.030, 1.446
Omphalitis (%)	0.462	0.271	1.71	0.094	−0.082, 1.006
Circulatory disorder (%)	−0.017	0.027	−0.64	0.526	−0.071, 0.037
Liver lesions (%)	−0.083	0.076	−1.08	0.283	−0.236, 0.071
Ascites (%)	−0.029	0.043	−0.67	0.504	−0.116, 0.058
Abnormal growth (%)	0.046	0.048	−0.97	0.338	−0.142, 0.050
Wounds (%)	−0.045	0.069	−0.65	0.517	−0.183, 0.093
Hock burns (%)	0.002	0.001	2.59	0.013	0.001, 0.004
Footpad dermatitis (%)	0.001	0.000	2.71	0.009	0.000, 0.002
Cleanliness score (0 to 300)	0.002	0.000	2.78	0.008	0.001, 0.003
Flock uniformity (CV)	−0.465	0.310	−1.50	0.140	−1.088, 0.158

a *z* is the standard score.

b CI is the confidence interval.

**Table 5:** Multiple linear associations of gait score in the broiler flocks

Gait score (log10)	Coefficient (log10)	SE	*t* ^a^	*P*>|*t*|	95% CI^b^
Peat litter *v*. wood shavings	0.087	0.029	3.01	0.004	0.029, 0.146
Live weight at visit (g)	−0.000	0.000	−2.49	0.017	−0.000, −0.000
Total condemnatio*n* (%)	0.054	0.018	2.91	0.006	0.016, 0.091
Hock burns (%)	0.004	0.001	4.99	0.000	0.002, 0.005
Cleanliness score (0 to 300)	0.003	0.001	5.14	0.000	0.002, 0.004

a *t* is the *t*-value of the *t*-test.

b CI is the confidence interval.

## Discussion

The aim of this study was to investigate the associations between lameness and commonly used health- and production-related measures of animal welfare in Norwegian broiler flocks. Briefly, in support of the hypothesis, a relationship between lameness and several of the health and production measures were identified. Thus, the results strongly support that there is a complex relationship between the overall flock health status, the production environment and lameness in broiler chickens. The results show that a substantial proportion (19%) of broilers displayed moderate-to-severe lameness (gait score ⩾3), which is in accordance with previous studies (Sanotra *et al.*, 2003; Knowles *et al.*, 2008; Bassler *et al.*, 2013; Kittelsen *et al.*, 2017). This indicates that potentially painful conditions are present on both individual and flock level, which may thereby compromise animal welfare (Vestergaard and Sanotra, 1999; Danbury *et al.*, 2000; Weeks *et al.*, 2000; Sanotra *et al.*, 2002; Caplen *et al.*, 2013; Hothersall *et al.*, 2016).

Although the prevalence of hock burns and footpad dermatitis were relatively low in the present study, lameness was strongly associated with increased prevalence of both hock burn and footpad dermatitis, which is in accordance with several earlier studies (De Jong *et al.*, 2014; Kittelsen *et al.*, 2017; Tullo *et al*., 2017). Severe scores of footpad dermatitis and hock burn have been associated with ulcerative and necrotic lesions on the broilers feet and hocks that may be painful (Haslam *et al.*, 2007; de Jong *et al.*, 2014). The evaluation of the severity of contact dermatitis lesions by histopathology has been recommended in previous reports, to validate the macroscopic scoring systems (Michel *et al.*, 2012; Zikic *et al.*, 2017). Since the scoring was performed in live birds, histology was not performed in the current study. The ulcerative lesions may be a gateway for bacteria, which could cause lameness in affected birds (Hester, 1994), as it is well known that cases of lameness may be associated with infectious components (Butterworth, 1999). Furthermore, we found both univariable and multivariable associations between increased lameness and dirtier birds (*P*<0.01), suggesting that cleanliness scoring should be considered as one potential indicator for the welfare of broilers. Lameness was not strictly coincident with the observed role of wet litter (*P*=0.07) in our study, which is well known to be associated with lameness from previous studies (de Jong *et al.*, 2014). Wet litter is a multifactorial problem that is affected by suboptimal ventilation, feed components, gut health, season, stocking density, litter depth and live weight (McIlroy *et al.*, 1987; Ekstrand *et al.*, 1998; Hermans *et al.*, 2006; Dunlop *et al.*, 2016). Several of these risk factors are directly linked to the management of the broiler production unit. Although results of the multiple regression show an association between litter type and lameness, the number of flocks using peat litter was only four (8%). It is, however, likely that litter type may influence on the litter quality, and thus being associated with lameness.

Lameness was associated with total condemnations at *postmortem* meat inspection, and with the subcategory ‘abnormal colour and odour’. Carcass condemnations due to abnormal colour and odour can result from septicaemia, toxaemia, poor bleeding or jaundice (Haslam *et al.*, 2008), but may occasionally include carcass appearances not strictly related to pathology. Since the percentage of birds condemned in this category was low in the present study, and since the underlying causes for the abnormal colour and odour in the condemned birds were not further investigated, the association with lameness needs further validation. No associations between flock mortality and lameness were observed in contrast to the association with 1^st^ week mortality found by Kittelsen *et al*. (2017).

Despite showing a weak effect, the live weight of the birds was inversely associated with gait score in this study. In addition, the growth rate was not statistically related to lameness, which is contradictory to other studies (Julian, 1998; Sanotra *et al.*, 2003; Kapell *et al.*, 2012). Although increased growth rate and increased live weight have been considered key factors for lameness in fast growing broilers (Julian, 1998; Kestin *et al.*, 2001; Sanotra *et al.*, 2003; Kapell *et al.*, 2012), high growth rates may also indicate an optimal physical environment in the house with a low infection pressure, and a farmer with good culling management. The variation of culling varied substantially between flocks in the present study, ranging from 0% to 55% of the total mortality, but there were no associations between culling and lameness. One reason for this may be that active culling reduces the number of lame birds in the flock by removal, and where culling rates are low, there may be a generally better health status, resulting in less lameness. The stocking density and age of the birds in the current study are lower than generally reported elsewhere, as maximum density in Norway is lower than EU (36 *v*. 42 kg/m^2^). There were no associations between lameness and stocking density in the present study. This contrasts previous studies where increased stocking density was identified as a risk factor for lameness in fast growing broilers (Sørensen *et al.*, 2000; Estevez, 2007). However, the environment provided for the birds can be more important than stocking density itself (Dawkins *et al.*, 2004; Jones *et al.*, 2005). Flock uniformity (often noted as CV) is a measure of how even the flock is, with regards to BW during lay (broiler breeders) or at slaughter (broilers), where a uniform flock is identified with a low CV (usually below 10%) (Petitte *et al.*, 1981; Feddes *et al.*, 2002). Poor uniformity may indicate reduced welfare, due to either management problems or health problems, including sub-clinical infections. Although there was a relatively large variation in flock uniformity in the 50 observed flocks (Table 1), we were unable to identify any association between flock uniformity and lameness in the present study. Further studies are needed to investigate associations between flock uniformity and health- and production measures.

In conclusion, 19% of the birds had a moderate-to-severe lameness, and lameness in the flock was associated with a range of health and production measures, including hock burns, footpad dermatitis, feather cleanliness and causes of condemnation. Although stocking density and growth rate are known key factors for lameness, our results suggest that a suboptimal physical environment may be another detrimental factor to leg health in addition to stocking density and growth rate. Since carcass condemnations, hock lesions and footpad dermatitis may be directly or indirectly associated with bacterial invasion, we suggest that future studies investigate the involvement of infections in lameness and other welfare outcomes.
